# Activity of a foam in preventing rebound of vancomycin-resistant *Enterococcus faecium*-containing droplets generated from the toilet bowl

**DOI:** 10.1007/s00203-023-03775-7

**Published:** 2024-01-08

**Authors:** Felice Valzano, Anna Rita Daniela Coda, Marianna Marangi, Gianfranco La Bella, Arcangelo Liso, Fabio Arena

**Affiliations:** 1 Department of Clinical and Experimental Medicine, University of Foggia, Via Napoli 20, 71122 Foggia, Italy; 2 Department of Medical and Surgical Sciences, University of Foggia, Via Napoli 20, 71122 Foggia, Italy; 3 Istituto Zooprofilattico Sperimentale Della Puglia E Della Basilicata, Via Manfredonia 20, 71121 Foggia, Italy; 4 IRCCS Don Carlo Gnocchi Foundation, Via Di Scandicci 269, 50143 Florence, Italy

**Keywords:** Vancomycin-resistant *Enterococcus faecium*, Multidrug-resistant bacteria, Hospital, Droplets, Toilet, Foam

## Abstract

In hospital environments, droplets generated by urination within shared toilets may represent a route of dissemination for bacteria such as vancomycin-resistant *Enterococcus faecium* (VREfm), which contributes significantly to the burden of hospital-acquired infections. We investigated the potential activity of a foam in preventing the generation of droplets containing *Enterococcus* spp. during urination. A uniform layer of foam was deposited in the inner walls and at the bottom of an experimental toilet contaminated with suspensions of *Enterococcus* strains (including a VREfm strain). Human urination was simulated, and colonies of *Enterococcus* were recovered through a toilet lid where agar plates had been placed. Results showed that the foam was able to suppress production of droplets containing *Enterococcus* spp. generated by a liquid hitting inner toilet walls. Conversely, *Enterococcus* colonies were recovered in absence of foam. Moreover, the foam did not show antibacterial activity. We propose a new non-antimicrobial approach aimed at limiting transmission of multidrug-resistant bacteria, particularly in healthcare settings.

**Graphical Abstract:**

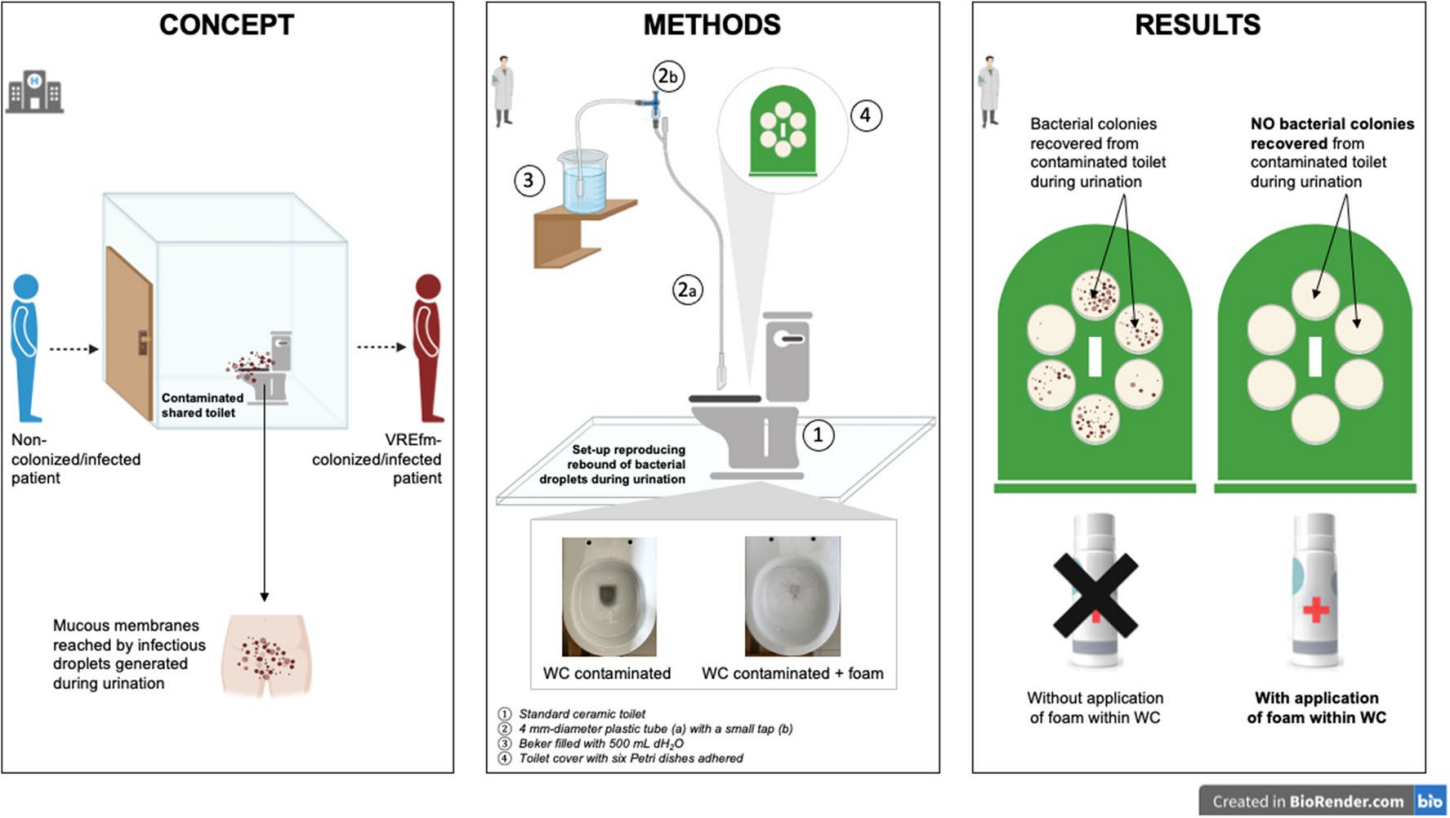

## Introduction

Vancomycin-resistant *Enterococcus faecium* (VREfm) represents an opportunistic pathogen in hospitalized patients with underlying diseases and the one most frequently isolated multidrug-resistant (MDR) pathogen in the healthcare setting (Arias and Murray 2012; Tacconelli et al. 2018). In addition to the broad intrinsic spectrum of drugs resistance, the emergence of vancomycin resistance mediated by *van*-type genes is cause of concern. VREfm infections are associated with prolonged hospitalization and higher mortality rates, with critically ill patients facing the greatest danger due to the limited treatment options (Prematunge et al. 2016; Geraldes et al. 2022).

The hospital environment offers optimal conditions for the transmission and spread of VREfm, with the contamination of room and/or bathroom playing a relevant role (Correa-Martinez et al. 2020). Transmission of MDR bacteria from these sources can occur through contact (i.e., touching), sprays and splashes, and inhalation (CDC 2019). Splashes may occur when water flow hits the contaminated drain cover or when a toilet or hopper is flushed. Splashes from the toilet can lead to dissemination of MDR-containing droplets, which in turn may contaminate the local environment or the skin of patients and nearby healthcare personnel (CDC 2019). Also, the production of droplets during urination can potentially contaminate the immediate area around the toilet and have a significant impact on the spread of bacterial pathogens.

Room contamination persists even after the adoption of standard cleaning procedures following discharge of VREfm-positive patients; this in turn, significantly increases the risk of new patients admitted to these rooms acquiring VREfm (Correa-Martinez et al. 2020). The adsorption of microorganisms to toilets porcelain may further facilitate VREfm persistence within toilet bowls after flushing (Verani et al. 2014; Johnson et al. 2017).

In this context, contaminated toilets may act as potential VREfm reservoir in the healthcare settings (Saliba et al. 2021), and consequently expose toilet users to infections through contact with hands or other parts of the body (e.g., human mucous membranes).

While the fact that VREfm has the capacity to adhere to various materials and to remain viable on environmental surfaces for several years is widely recognized (Suleyman et al. 2018), studies evaluating the potential spreading of this organism through toilets are lacking. Recently, we demonstrated the formation of rebound droplets containing multidrug-resistant *Klebsiella pneumoniae* in a model simulating female urination (Arena et al. 2021).

In this study, we evaluated the dispersion of droplets containing bacteria belonging to the genera *Enterococcus* potentially generated following simulation of the urine flow hitting the toilet bowl walls. In addition, we investigated the potential activity of a new foam composition in preventing the dispersion of such droplets.

## Materials and methods

### Bacterial strains

The study included the vancomycin-susceptible reference strain *E. faecalis* ATCC 29212, an isolate from human urine (Kim et al. 2012), and a vancomycin-resistant *Enterococcus faecium* (VREfm) clinical isolate (i.e., the VREfm12 strain), which was positive for *vanA* gene and obtained from blood cultures of a patient with sepsis in 2022.

Identification was performed by matrix-assisted laser desorption ionization–time of flight mass spectrometry (MALDI-ToF MS) (Bruker Daltonics, Billerica, USA). Antimicrobial susceptibility was determined by broth microdilution using commercial plates (ITGP MICRONAUT-S plates, Merlin Diagnostika GmBH, Germany). Antimicrobial susceptibility testing results were read after 18 ± 2 h of incubation at 35 ± 1 °C in aerobic atmosphere and interpreted according to EUCAST clinical breakpoints v. 13 (EUCAST 2023).

The presence of *van* genes (*vanA*, *vanB*) was determined in a rapid isothermal amplification reaction, which was performed using the Amplex assay eazyplex VRE kit (AmplexDiagnostics GmbH, Werkstrasse, Germany).

For the preparation of the bacterial cultures used in the experiments, the strains were cultured onto tryptic soy agar (TSA) (Oxoid, Milan, Italy) at 35 ± 2 °C in ambient air overnight.

### Experimental toilet device

**Fig. 1:**
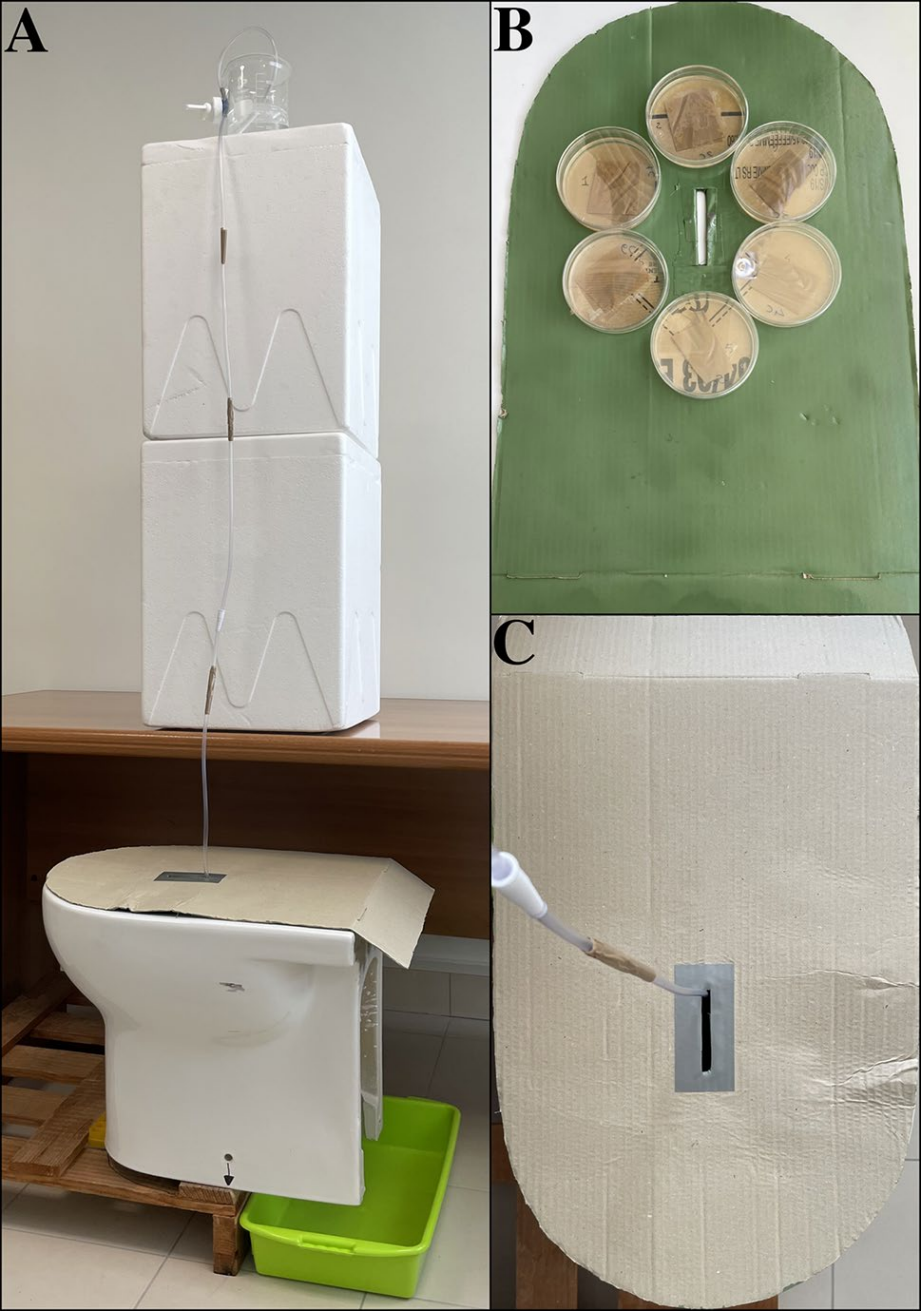
Experimental equipment used to reproduce the rebound of bacterial droplets during urination. **A** A standard ceramic toilet and a beaker containing 500 mL of sterile distilled water passed through a 4 mm internal diameter tube; **B**, **C** a toilet cover, with attached six TSA plates (positioned toward inner side of the toilet) and having a slot through which to pass the tube to reproduce urination of a person who is sitting

Human urination was simulated using the experimental toilet device previously reported by Arena et al. (2021) (Fig. 1). Briefly, the equipment included a standard ceramic toilet bowl with a device consisting of a 4 mm diameter plastic tube, a beaker containing 500 mL of sterile distilled water positioned at a height of 2 m and a small tap to regulate the flow of water to pass through the tube. The hydrodynamics of human urination was mimicked using a jet of liquid at 37 °C with a flow rate of ~ 20 mL/s. A cardboard lid with six Petri dishes of TSA (Oxoid) applied to the side facing the inside of the toilet was used for the recovery of bacterial colonies after urination. The tube was placed through a small slit in the toilet cover to reproduce a person sitting while urinating (Fig. 1).

We chose to mimic human urination rather than using human urine since the latter is considered a non-sterile human fluid (Wolfe and Brubaker 2015). Consequently, the potential use of human urine could have represented a contamination source in our experiments.

### Foam composition

The aqueous foam composition (international patent application No. WO2021/198837A1) includes the following chemicals: a betaine (i.e., alkylamidopropyl betaine) as emulsifying and stabilizing agent; an anionic surfactant (i.e., sodium salt of alkyl sulphate) with detergent and foaming properties; a dialkyl carbonate (i.e., bis-propylheptyl carbonate) exerting both a modulatory activity on pH and contributing to foam consistence; a mixture of fatty alcohols (i.e., acetyl alcohol and stearyl alcohol) and a fatty acid ester, both contributing to foam structure; a pH regulator (i.e., lactic acid); a mixture of deodorizing substances (e.g., triethyl citrate, bergamot essence); and a propellant gas for the delivery of the foam.

The foam is also characterized by (1) specific persistence and adhesion properties, (2) biodegradability for environmental sustainability, (3) stability regardless of the temperature, humidity and ventilation conditions, (4) constant coverage and visibility of the deposited surface, (5) easy elimination with the standard quantity of water used for toilet flushing, and (6) a pleasant appearance and smell.

The foam was delivered using a practical spray that allowed it to be deposited into the bowl as thick layer (2–3 cm approximately) by manually orienting the nozzle.

### Evaluation of *Enterococcus* spp. droplets rebound

The potential rebound of droplets containing *E. faecalis* ATCC 29212 and *E. faecium* VREfm12 was evaluated with and without the application of foam as described previously (Arena et al. 2021), with minor modifications. Briefly, after overnight growth on TSA (Oxoid), six or seven colonies for each strain were suspended in saline solution for the preparation of the 0.5 McFarland standard. This suspension was then diluted in 1500 mL of sterile distilled water to reach a final bacterial concentration of ~ 1.5 × 10^6^ CFU/mL, potentially present in toilet water and wastewater (Barker and Bloomfield 2000; Ottoson and Stenström 2003; Oteng-Peprah et al. 2018). Before and after each experiment, the toilet surfaces were manually washed with 70% ethanol and subsequently with sterile distilled water. During the urine flow simulation, the tube was positioned orienting the water jet for 15 s against the front wall of the toilet and for another 15 s against the back wall, with a flow rate of ~ 20 mL/s (i.e., resembling the hydrodynamics of human urination).

The experiments involved three experimental conditions: (1) control (i.e., not contaminated toilet), (2) no foam (i.e., contaminated toilet without foam), and (3) foam (i.e., contaminated toilet with the application of foam). In the first condition, 1500 mL of sterile distilled water were deposited into the toilet thoroughly wetting all the internal walls and filling the bottom. The toilet seat was positioned with six TSA plates facing the inner side of the toilet and the liquid jet was produced with 500 mL of sterile distilled water at 37 °C for 30 s. For the second condition (no foam), the inner surfaces of the toilet and the bottom were contaminated with 1500 mL of solution containing a final bacterial concentration of ~ 1.5 × 10^6^ CFU/mL. Afterwards, the same procedure used in the first condition to recover bacterial cells was performed. For the third condition (foam), the toilet contamination was immediately followed by the deposition of a uniform foam layer (~ 2–3 cm thickness) on the inner walls of the toilet bowl and inside air–liquid interface. Finally, the liquid jet was produced with 500 mL of sterile water for 30 s.

The mean number of CFU/cm^2^ was then determined incubating TSA plates for 24 h at 35 °C. The colony count was also double-checked after 48 h of incubation. Representative recovered colonies for each plate were identified to species level by MALDI-TOF MS technology (Bruker Daltonics).

Data were obtained in at least three independent experiments, with one replicate per condition per experiment.

**Fig. 2:**
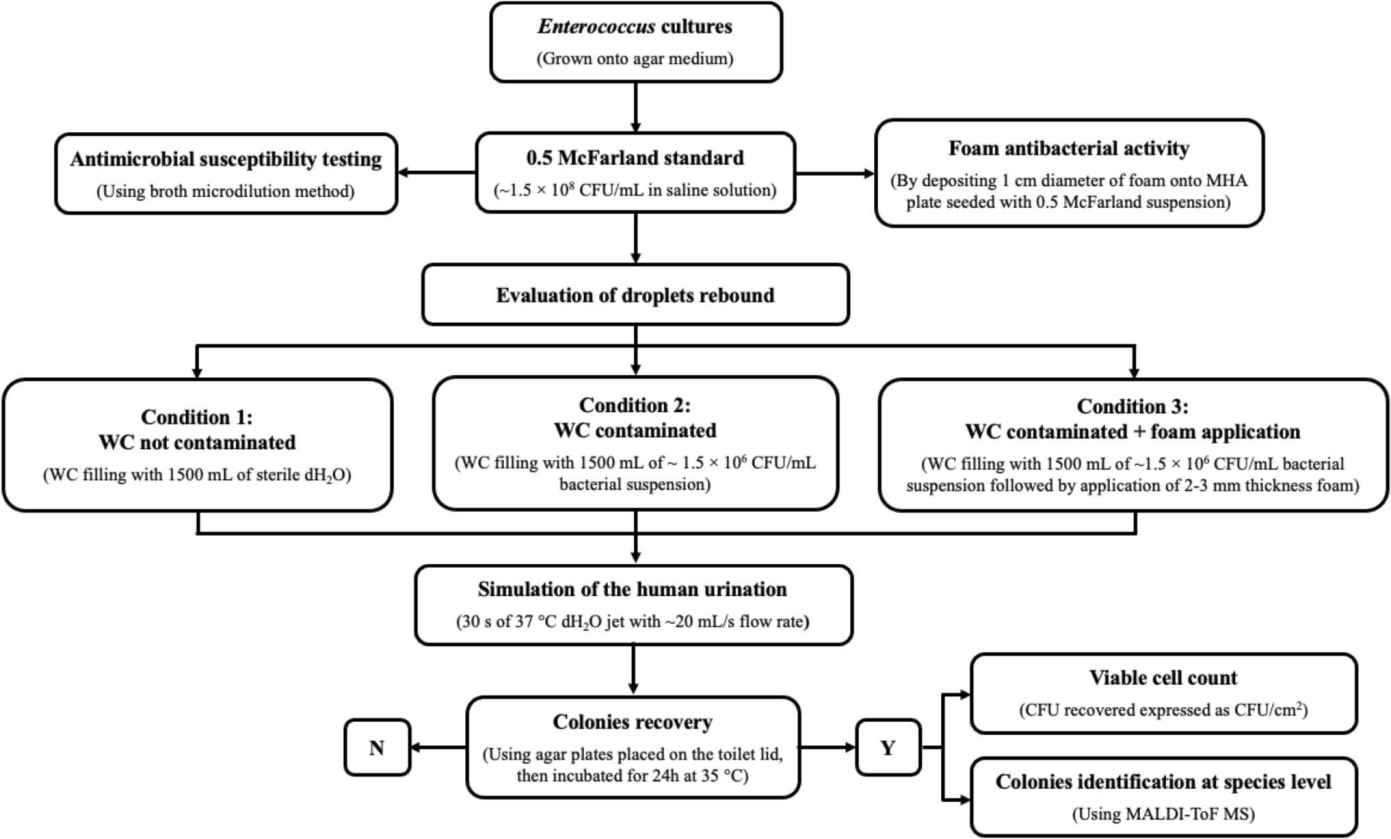
Schematic diagram illustrating the experimental workflow of the study. CFU colony forming unit, MHA Mueller–Hinton agar, MALDI-ToF MS matrix-assisted laser desorption ionization–time of flight mass spectrometry

A schematic representation of the aforementioned steps is shown in Fig. 2.

### Foam antibacterial activity testing

The in vitro antibacterial activity of the foam was carried out with both the strains included in the study (i.e., *E. faecalis* ATCC 29212 e *E. faecium* VREfm12). Briefly, a standard bacterial suspension (i.e., 0.5 McFarland) was homogeneously spread onto Mueller–Hinton agar (MHA) plates with a thickness of ~ 4 mm and a small amount of foam (~ 1 cm in diameter) deposited in the center of the plates (Fig. 2). After 18–24 h at 35 ± 2 °C, the plates were visually inspected for the presence or absence of growth inhibition in the area where the foam was applied. The experiments were performed in at least three independent experiments, with three replicates per experiment.

### Statistical analysis

Statistical analysis was performed using GraphPad Prism version 8.0 (San Diego, CA, USA). Unpaired *t* test with Welch’s correction was used for comparisons between data obtained with and without foam.

## Results

### Rebound droplets containing *Enterococcus* spp. generated during simulated urination

The generation of droplets containing *Enterococcus* spp. during simulated urination was evaluated in a contaminated toilet with bacterial suspensions of *E. faecalis* ATCC 29212 and *E. faecium* VREfm12.

**Fig. 3:**
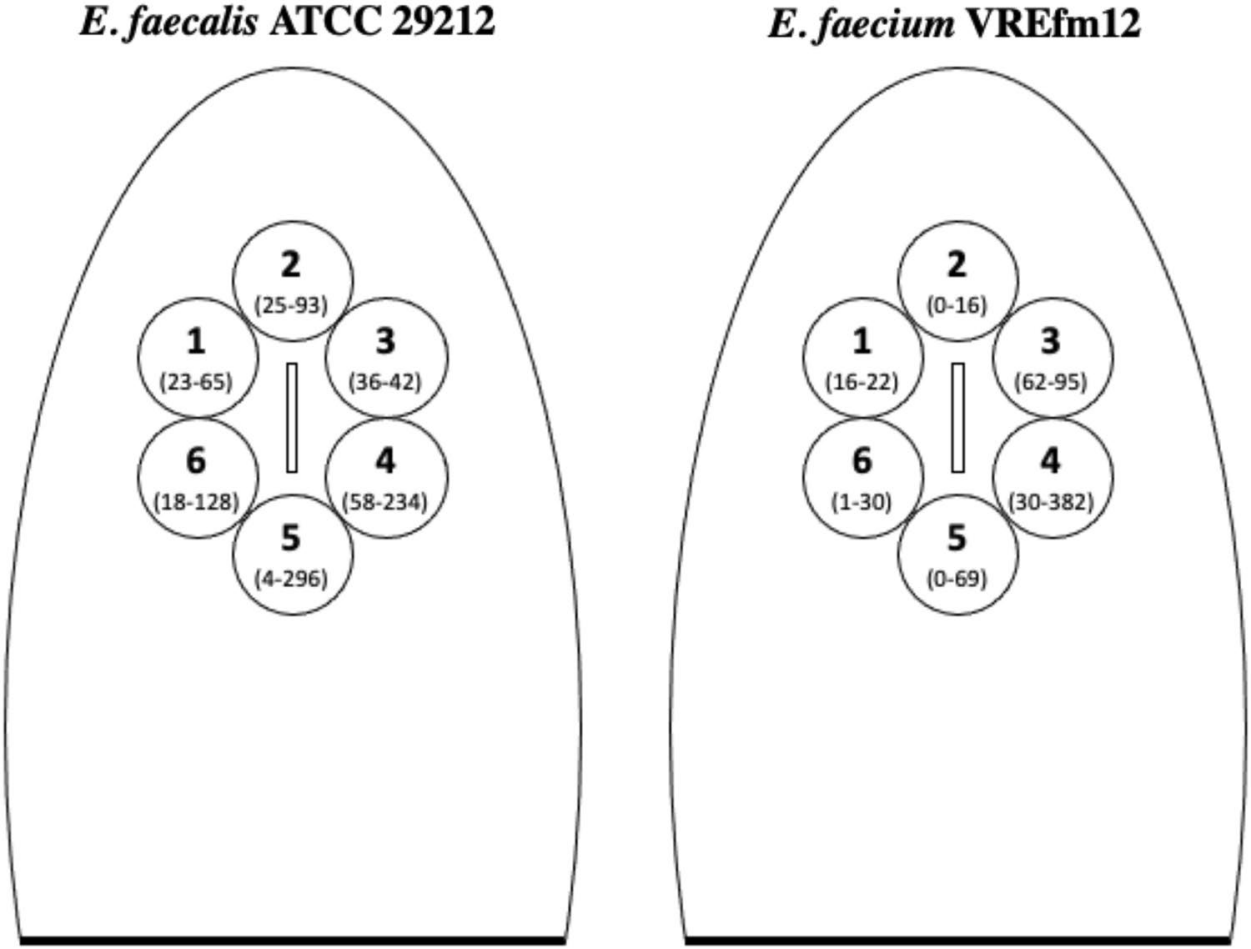
Schematic reproduction of the toilet seat with the Petri dishes numbered from 1 to 6. For each plate, a numerical range is reported in brackets, indicating the number of bacterial colonies of *E. faecalis* ATCC 29212 and *E. faecium* VREfm12 recovered, expressed as colony forming units (CFU), in the condition where the foam was not used throughout the three experiments.

**Fig. 4:**
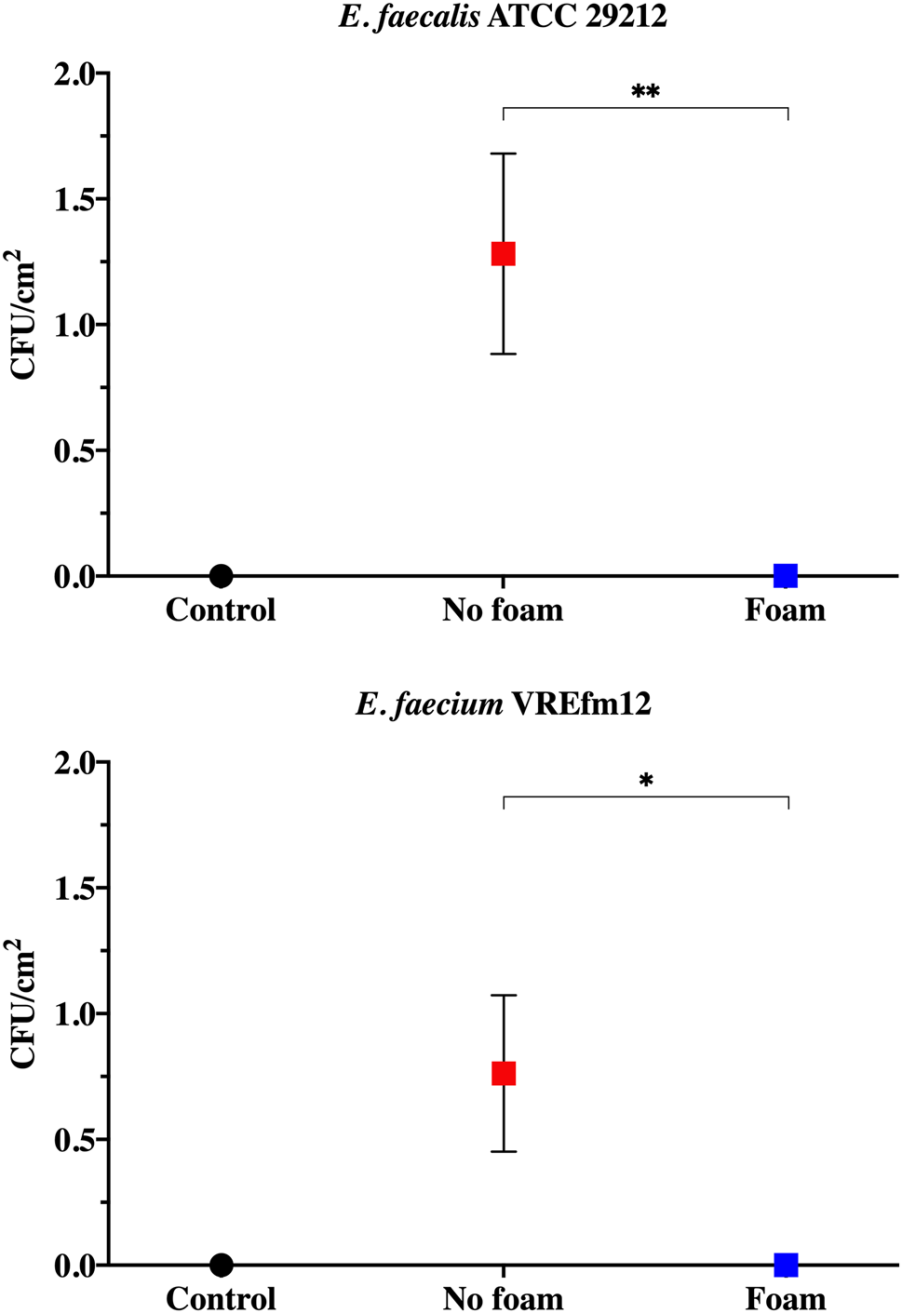
CFU/cm^2^ of *E. faecalis* ATCC 29212 and *E. faecium* VREfm12 recovered with and without foam. Recovered colonies were expressed as mean and standard error of mean of CFU/cm^2^, normalized for the total area of the six Petri dishes in the three experimental replicates (*p < 0.05, **p < 0.01). Black dot represents control (i.e., the toilet not contaminated) while blue and red squares represent the conditions with and without foam application, respectively

Overall, we observed a substantial recovery of bacterial colonies of both tested strains following simulation of urination, with a range of 4–296 and 0–382 CFU of *E. faecalis* ATCC 29212 and *E. faecium* VREfm12 recovered among all plates, respectively (Fig. 3). In particular, the mean number of bacterial colonies recovered was 1.28 ± 0.40 and 0.76 ± 0.31 CFU/cm^2^ (i.e., mean value ± standard error of mean) for *E. faecalis* ATCC 29212 and *E. faecium* VREfm12, respectively, highlighting a probable strain or species-dependent difference of the number of bacterial cells recovered (Fig. 4). Notably, we found a trend towards a higher recovery of colonies in the back side of the toilet (i.e., Petri dishes n. 4, 5 and 6) as compared to the front side (i.e., Petri dishes n. 1, 2 and 3), regardless of the strain tested (Fig. 3).

### Foam-mediated inhibition of *Enterococcus* spp.-containing droplets rebound generated during simulated urination

The potential activity of the foam in preventing the rebound of droplets generated during simulated urination was investigated against both reference strain (i.e., *E. faecalis* ATCC 29212) and VREfm clinical isolate (i.e., *E. faecium* VREfm12). After the toilet contamination, urination was simulated in the presence of a foam layer (~ 2–3 mm thickness) deposited immediately before on the inner walls and at the air–liquid interface of the toilet.

A complete suppression of infectious droplets production was observed in the presence of foam within toilet contaminated with both *E. faecalis* ATCC 29212 and *E. faecium* VREfm12. In fact, no growth was detected in Petri dishes used in the condition “foam” for each tested strain (Fig. 4). This activity can be connected to the mechanical effect exerted by the foam against rebounding droplets trapping them and preventing their dispersion beyond the toilet bowl.

### Antibacterial activity of the foam

**Fig. 5:**
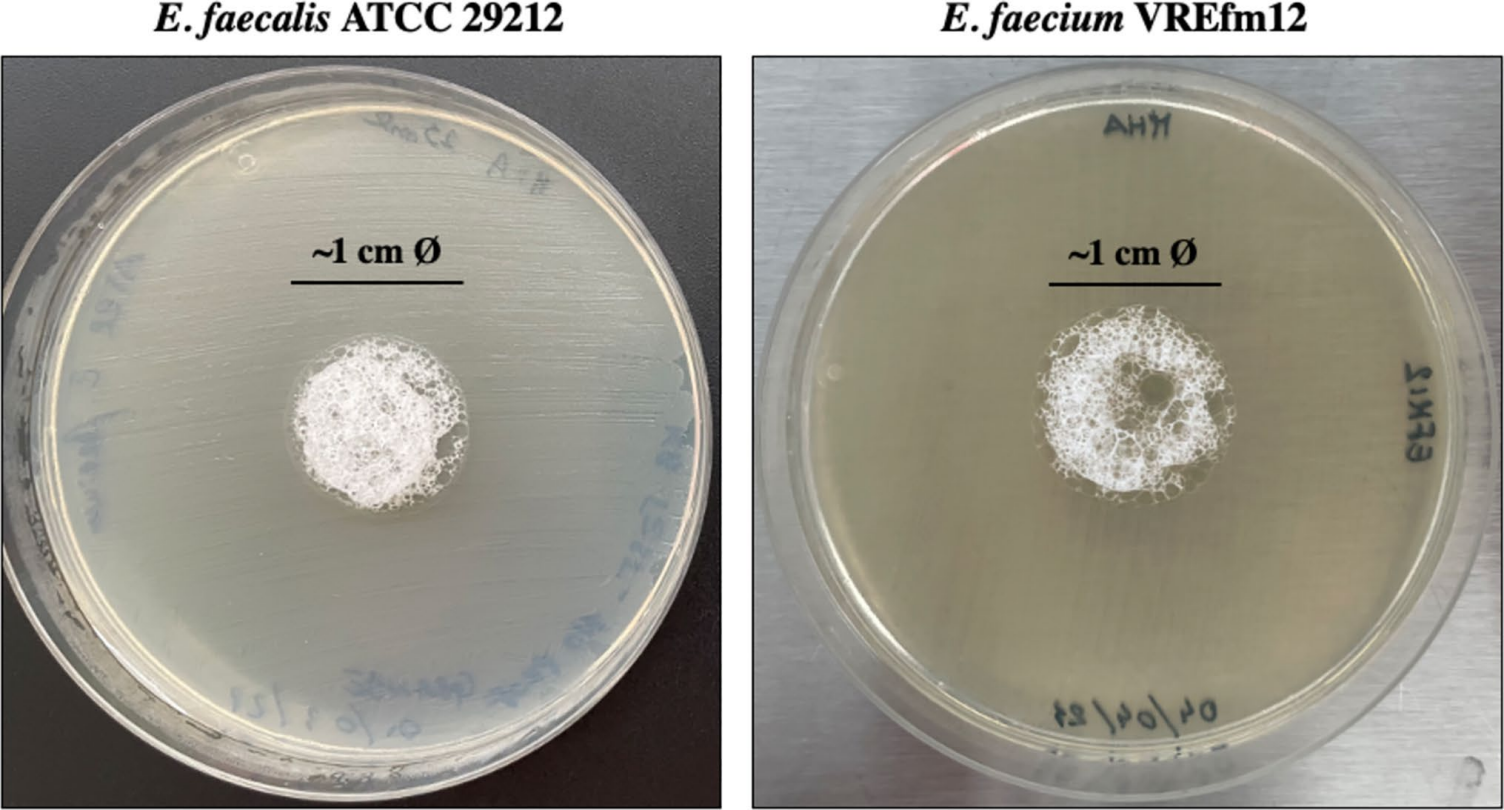
Antibacterial activity of the foam against *E. faecalis* ATCC 29212 and *E. faecium* VREfm12. Assays were performed in at least three independent experiments (with three replicates per experiment), and representative data are shown

After the incubation period, no growth inhibition was observed in the zone of the Petri dish where foam was present (Fig. 5).

These results demonstrate that the activity of the foam is probably related to a mechanical effect rather than a biological one, supporting the hypothesis that the foam would trap droplets generated during urination within toilet bowl, thus preventing their rebound.

## Discussion

The ability of microorganisms to persist within toilet bowl despite the use of the toilet flush, is a common phenomenon widely reported elsewhere (Gerba et al. 1975; Johnson et al. 2013, 2017). The toilets hence represent an important reservoir of bacterial pathogens, which may spread through infectious bioaerosol and/or droplets generated following flushing and during urination (Gerhardts et al. 2012; Grabowski et al. 2017; Arena et al. 2021; Hamerlinck et al. 2023), potentially leading to an indirect transmission of infections. This is particularly relevant in hospital and healthcare settings, where toilets are used by patients who are infected or colonized by pathogenic microbes.

In this respect, stools and urine from patients carrying multidrug-resistant (MDR) bacteria may be a source of resistance determinants, which spread in the environment and represent a serious concern for patient clinical outcomes (Kizny Gordon et al. 2017; Abney et al. 2021). Among MDR bacteria colonizing the gut of inpatients, vancomycin-resistant *Enterococcus faecium* (VREfm) plays an important role, and it is associated with higher rates of contamination comparing to other MDR organisms (Saliba et al. 2021). Moreover, VREfm is more frequently involved in nosocomial transmission events than other relevant resistant bacteria (Erb et al. 2017). The isolation of patients colonized or infected by MDR organisms, coupled with the implementation of rigorous cleaning procedures, yields only partial effectiveness in interrupting the chain of transmission (Eckstein et al. 2007; Kluytmans-van den Bergh et al. 2019; Maechler et al. 2020). This is possibly due to the absorption of bacteria to toilets porcelain (Johnson et al. 2017). In fact, it has been reported that hospital toilets represent a transmission vector for VREfm, being directly involved in the transmission of this microorganism (Noble et al. 1998; Cassone et al. 2020; Correa-Martinez et al. 2020). Consequently, VREfm contamination and persistence within the toilet bowl represent a real risk factor for VREfm-negative patient colonization.

For this reason, additional measures for preventing VREfm acquisition should be considered.

In this study, we demonstrated that rebound droplets containing both *E. faecalis* ATCC 29212 and *E. faecium* VREfm12 are generated by a liquid jet mimicking human urination and hitting the inner walls of a contaminated toilet, thus representing a potential route of environmental *Enterococcus* contamination. In particular, the genitourinary mucosa and the perineal cutaneous surface, especially those of people urinating while sitting, can be reached by these droplets. Interestingly, an innovative foam composition applied to the interior walls of the toilet before simulated urination has been found to effectively eliminate droplet rebound. In fact, *Enterococcus* colonies were not recovered on any plate under such a condition, confirming what was previously reported with a carbapenem-resistant *Klebsiella pneumoniae* strain (Arena et al. 2021). This phenomenon is probably due to the mechanical activity of the foam, which prevents the dispersion of bacterial droplets outside the toilet by trapping them.

Our model simulates a VREfm heavy contamination of the internal toilet bowl to resemble the microbial concentrations possibly observed in wastewater (Barker and Bloomfield 2000; Ottoson and Stenström 2003; Oteng-Peprah et al. 2018). Consequently, the evidence that foam prevents the generation of droplets containing Gram-positive bacteria (such as *Enterococcus* strains) in addition to what was already published with the same tool, allows to confirm its role in limiting the spread of infections caused by MDR bacteria, especially within healthcare environments.

Furthermore, our results showed that the foam does not inhibit the growth of the VREfm strain (i.e., a representative strain of the resistance pattern mainly observed in Italy for VREfm of clinical relevance), which potentially means an absence of antibacterial activity of the foam composition. This is particularly important as it would prevent the development of resistance to chemical compounds and related antibiotics. However, testing antimicrobial activity of each foam component would be necessary to fully support our finding.

Despite the unequivocal effect exerted by the foam, there are some limitations to the present study. We investigated only a type of toilet in detail and thus only one design. In this regard, we noticed that colonies recovery, in the experiments performed without the foam, were higher in the plates placed in the back side of toilet cover. We speculate that this finding is related to the geometry of the inner toilet walls which indeed can affect the rebound of droplets. For this reason, further studies involving different toilet designs will be needed to support our findings. Moreover, we simulated urination of a person who is sitting, whose mucous membranes are closer to the toilet walls (and water) than a person who is standing. More experiments simulating urination at different height are needed to confirm our findings.

Overall, we propose that, in toilets and especially those in hospitals, may be important to consider the use of physical tools such as foam compositions, which along with chemical sanitization may help to reduce the risk of infection.

Finally, in the future, the activity of other foam compositions containing antibacterial chemical compounds can be investigated.

## Conclusions

In conclusion, the results of this study demonstrate a relevant activity of the foam in preventing the generation of droplets containing *Enterococcus* strains in the toilet (including droplets containing a VREfm clinical isolate). Compared to previously published studies this investigation represents an advance because it provides new data on the foam-mediated inhibition of rebound droplets containing Gram-positive bacteria.

In addition, in vitro antibacterial activity testing reveals that the foam does not show an antimicrobial activity against strains included in the study, which can be very relevant in terms of prevention of resistance development.

Our findings suggest that foam-based products can have a potential role in limiting the spreading of multidrug-resistant microorganisms in shared restrooms, especially those present in healthcare settings.

## Data Availability

The authors confirm that the data supporting the findings of this study are available within the article.
